# Intracoronary physiology-guided percutaneous coronary intervention in patients with diabetes

**DOI:** 10.1007/s00392-023-02243-y

**Published:** 2023-06-20

**Authors:** Roberto Scarsini, Matteo Tebaldi, Francesca Rubino, Sara Sgreva, Giovanni Vescovo, Marco Barbierato, Andrea Vicerè, Domenico Galante, Concetta Mammone, Mattia Lunardi, Domenico Tavella, Gabriele Pesarini, Gianluca Campo, Antonio Maria Leone, Flavio Luciano Ribichini

**Affiliations:** 1 Division of Cardiology, Department of Medicine, Verona University Hospital, Piazzale A. Stefani 1, 37126 Verona, Italy; 2 Azienda Ospedali Riuniti Marche Nord, Emodinamica e Cardiologia Interventistica, Pesaro, Italy; 3 Ospedale dell’Angelo, Mestre, Italy; 4 Istituto di Cardiologia Università Cattolica del Sacro Cuore, Rome, Italy; 5 Dipartimento di Scienze Cardiovascolari Fondazione Policlinico Universitario A. Gemelli IRCCS, Rome, Italy; 6 Diagnostic and Interventional Unit Ospedale Fatebenefratelli Isola Tiberina Gemelli Isola, Rome, Italy; 7 Cardiology Unit, Azienda Ospedaliero Universitaria di Ferrara, Cona (Ferrara), Italy; 8 Division of Cardiology, University of Verona, Piazzale A. Stefani 1, 37126 Verona, Italy

**Keywords:** Diabetes mellitus, Insulin, Fractional flow reserve, Instantaneous wave-free ratio, Coronary artery disease

## Abstract

**Objective:**

The risk of vessel-oriented cardiac adverse events (VOCE) in patients with diabetes mellitus (DM) undergoing intracoronary physiology-guided coronary revascularization is poorly defined. The purpose of this work is to evaluate the risk of VOCE in patients with and without DM in whom percutaneous coronary intervention (PCI) was performed or deferred based on pressure-wire functional assessment.

**Methods:**

This is a retrospective analysis of a multicenter registry of patients evaluated with fractional flow reserve (FFR) and/or non-hyperaemic pressure ratio (NHPR). Primary endpoint was a composite of VOCE including cardiac death, vessel-related myocardial infarction (MI), and ischemia-driven target vessel revascularization (TVR).

**Results:**

A large cohort of 2828 patients with 3353 coronary lesions was analysed to assess the risk of VOCE at long-term follow-up (23 [14–36] months). Non-insulin-dependent-DM (NIDDM) was not associated with the primary endpoint in the overall cohort (adjusted Hazard Ratio [aHR] 1.18, 95% CI 0.87–1.59, *P* = 0.276) or in patients with coronary lesions treated with PCI (aHR = 1.30, 95% CI 0.78–2.16, *P* = 0.314). Conversely, insulin-dependent diabetes mellitus (IDDM) demonstrated an increased risk of VOCE in the overall cohort (aHR 1.76, 95% CI 1.07–2.91, *P* = 0.027), but not in coronary lesions undergoing PCI (aHR 1.26, 95% CI 0.50–3.16, *P* = 0.621). Importantly, in coronary lesions deferred after functional assessment IDDM (aHR 2.77, 95% CI 1.11–6.93, *P* = 0.029) but not NIDDM (aHR = 0.94, 95% CI 0.61–1.44, *P* = 0.776) was significantly associated with the risk of VOCE. IDDM caused a significant effect modification of FFR-based risk stratification (*P* for interaction < 0.001).

**Conclusion:**

Overall, DM was not associated with an increased risk of VOCE in patients undergoing physiology-guided coronary revascularization. However, IDDM represents a phenotype at high risk of VOCE.

**Graphical abstract:**

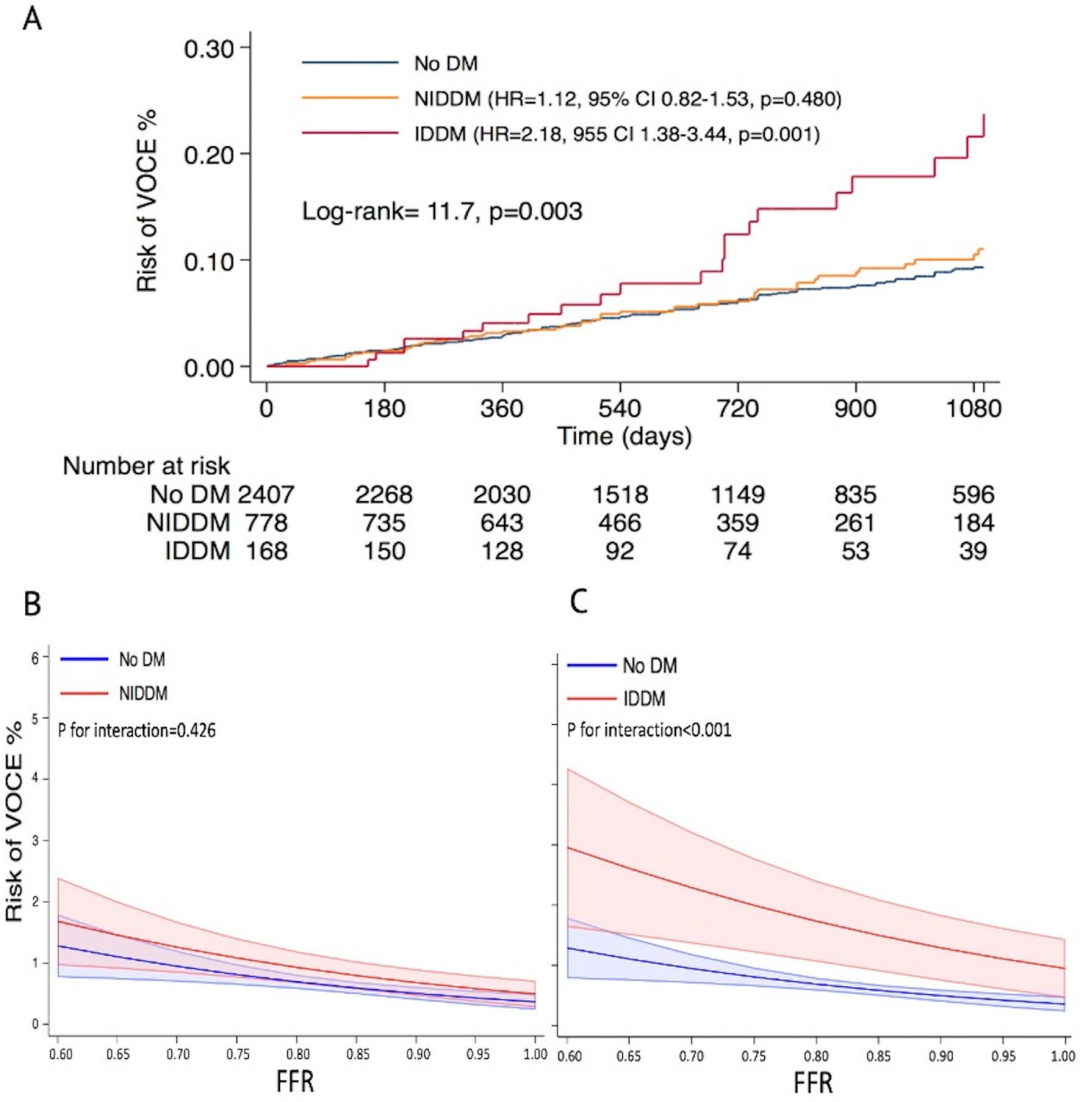

## Introduction

Intracoronary physiology assessment of intermediate severity coronary artery disease (CAD) is recommended in patients without non-invasive evidence of inducible ischemia and in patients with the multivessel disease [1, 2]. However, the reliability of pressure-wire-based evaluation is still debated in specific clinical settings including diabetes mellitus (DM). Indeed, in patients with DM, the frequent association of coronary microvascular dysfunction and vulnerable plaque features may hamper the accuracy of intracoronary functional assessment [3]. On the other hand, the advantages offered by physiology-guided intervention may be particularly relevant in patients with DM considering that (1) percutaneous coronary intervention (PCI) yields inferior long-term results in patients with DM compared with non-diabetic patients [4]; (2) coronary revascularization offers scarce advantages over medical therapy in diabetic patients [5, 6]; (3) patients with DM tend to show a more aggressive and diffuse atherosclerotic disease with frequent multivessel involvement. However, if DM is associated with an increased risk of vessel-oriented adverse cardiovascular events (VOCE) in patients undergoing coronary physiology assessment remains poorly defined. In this study, we aimed to assess the risk of VOCE in long-term in patients with and without DM who underwent physiology-guided coronary revascularization. Moreover, we aimed to identify clinical features associated with an increased risk of adverse outcomes among patients with DM, particularly when coronary intervention was deferred based on intracoronary functional assessment.

## Methods

This is a retrospective analysis based on a large multicenter registry of patients who underwent pressure-wire-based coronary functional assessment at 4 major cardiovascular interventional centers in Italy (Verona University Hospital, Verona; Policlinico Agostino Gemelli, Rome; Ferrara University Hospital, Ferrara; Ospedale dell’Angelo, Mestre).

**Fig. 1:**
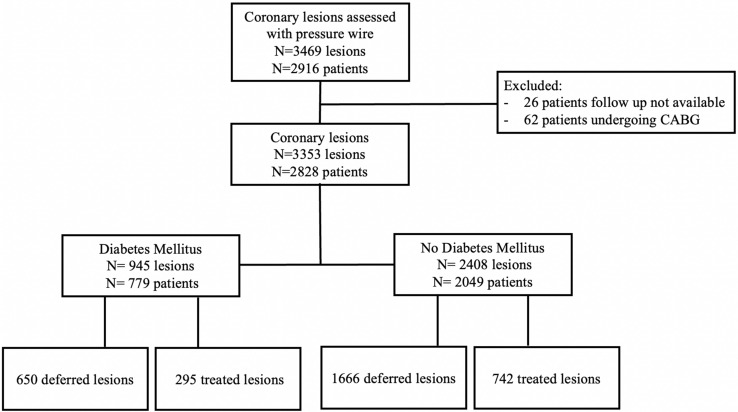
Study flowchart

Patients with and without DM with at least one intermediate coronary lesion evaluated with fractional flow reserve (FFR) and/or non-hyperaemic pressure ratios (NHPR) were included in the analysis (Fig. 1). Patients with previous coronary artery bypass graft surgery (CABG), severe aortic stenosis and clinical follow-up not available were excluded. Moreover, culprit vessels of recent (< 30 days) ST-segment elevation acute coronary syndrome were also excluded. Patients undergoing CABG after the index coronary angiography with functional assessment procedure were excluded from further analysis. (Fig. 1, Supplementary Table 1)

Diagnosis of DM, insulin-dependent diabetes mellitus (IDDM), arterial hypertension and dyslipidaemia were determined based on information collected from patients or medical records by the investigating physicians. Patients with impaired fasting glucose were considered nondiabetic. CKD was defined as an estimated glomerular filtration rate < 60 ml/min/1.73 m^2^ estimated using the Cockroft-Gault equation. Target organ damage was defined as severe renal impairment (eGFR < 30 ml/min/1.73 m^2^) and/or severe target organ vasculopathy (including multivessel coronary disease, carotid artery disease or peripheral vascular disease) [7, 8].

The study was conducted according to the Declaration of Helsinki and approved by the institutional review board of each participant centres. All the patients provided their informed written consent to the anonymous data collection. All authors contributed to the production of the manuscript: RS, FLR, FR and SS conceived and designed the study, interpreted the data, and drafted the manuscript; MT, GV, AV, DG, CM, ML, RS, FR and SS collected and analyzed the data; MT, GV, MB, DT, GP, GC, AML and FLR revised the manuscript critically for important intellectual content.

### Intracoronary functional assessment

Functional assessment of coronary lesions was performed using standard pressure-wire technology (Pressure-wire X Abbott Vascular, Santa Clara CA, or Prestige Plus or Verrata Pressure Wire, Philips, The Netherlands). Intracoronary nitrates (200–300 mg) were administered before performing any physiological measurement. The choice of the physiological index for the assessment of CAD and the decision on treatment were based on the operators’ clinical judgment. FFR was defined as the ratio between distal coronary pressure (Pd) and aortic pressure (Pa) under steady-state hyperaemia. Hyperaemia was obtained using an intravenous infusion of adenosine (140 mg/kg/min) or an intracoronary bolus of 150–250 ug of adenosine. Among NHPRs, Pd/Pa was measured during the full cardiac cycle, whereas the instantaneous wave-free ratio (iFR) was defined as the lowest Pd/Pa measured during the diastolic wave-free period using a dedicated commercial software (Philips, The Netherlands). FFR value ≤ 0.80 and NHPRs ≤ 0.89 were considered abnormal, as recommended [1]. In 17.8% of the lesion, both FFR and NHPR were available. In the case of FFR/NHPR discordance, coronary physiology was defined “abnormal” if FFR was ≤ 0.80.

### Study endpoints and adverse clinical events definition

The primary endpoint was the composite of VOCE including ischemia-driven target vessel revascularization (TVR), vessel-related myocardial infarction (MI), and vessel-related cardiovascular death at the longest follow-up time available. The secondary endpoints were the individual components of the primary endpoint. Clinical follow-up was obtained through the hospital clinical records at the date of death or at the last outpatient visit. When data were not available, follow-up was obtained through telephone contacts. Physicians collecting clinical follow-up data were unaware of the study design. All events were adjudicated by independent operators at each interventional site. Events were designated as vessel related or not vessel related. The adverse events were defined as follows: MI was defined as readmission with a primary diagnosis of non–ST-segment elevation MI or ST-segment elevation MI at any time after the index procedure according to the 4th universal definition of MI [9]. Any MI without a clearly identifiable culprit vessel was counted as target vessel related. Revascularization was defined as any unplanned percutaneous or surgical revascularization of the coronary vessel originally evaluated by pressure-wire assessment. All deaths were considered cardiovascular unless an unequivocal noncardiac cause could be established. Cardiovascular death in patients with multiple diseased vessels was assigned to each vessel.

### Statistical analysis

Categorical variables are expressed as number and percentages. Continuous variables are presented as mean ± standard deviation (SD) or median and interquartile range (IQR) as appropriate. Comparisons between continuous variables were performed using the Student’s *t* test or Mann–Whitney *U* test, as appropriate. Comparisons between categorical variables were evaluated using Fisher’s exact test or Pearson’s chi-square test, as appropriate.

Survival analysis was performed using Kaplan–Meier plots and differences between groups were estimated using the log-rank test. Cox proportional regression analysis was performed to estimate hazard ratios (HR). Variables with a level of significance < 0.10 at univariable analysis were included in the multivariable Cox regression models and 95% confidence intervals of the HRs were provided. The test for proportional-hazards assumption was applied to confirm the validity of the model. Shared frailty Cox regression multivariable analysis, with patient identification introduced in a multilevel model, was performed to take into account the nonindependence of lesions. Interaction analysis was used to assess the effect modification of different variables on the primary endpoint. A *P*-value ≤ 0.05 was considered significant. Statistical analyses were performed using Stata (Stata Corp., 2018) and SPSS 26.0 software (IBM Inc., New York, USA).

## Results

### Study population

Two-thousand-nine-hundred-sixteen patients with 3469 coronary lesions of intermediate angiographic severity underwent coronary physiology assessment were included in this study. Long-term clinical follow-up was available for 2828 patients and 3353 coronary lesions (Fig. 1). The median follow-up time was 23 months (IQR 14–36 months). DM was present in 779 (27.5%) patients with 945 (28.2%) coronary lesions. Among patients with DM, 81.6% had non-insulin-dependent DM (NIDDM) and 18.4% had IDDM. Clinical and angiographic characteristics of the study cohort were reported in Table 1. FFR was measured in 2968 lesions (88.5%) coronary vessels. Both FFR and NHPRs were available in 597 (17.8%) vessels. Conversely, NHPRs alone were measured in 385 (11.5%) vessels (Supplemental Fig. 1). Sixty-two patients with 90 lesions assessed with intracoronary physiology underwent CABG surgery and were excluded from further analysis (Fig. 1, Supplementary Table 1). Coronary revascularization with PCI was performed in 1037 coronary lesions (30.9%) and it was deferred in 2316 (69.1%) lesions.

**Table 1:** Clinical and angiographic characteristics of coronary lesions of patients without DM, with DM non-insulin-dependent and with IDDM

	No-DM (A)	NIDDM (B)	IDDM (C)	*P*-value A vs B	*P*-value A vs C	*P*-value B vs C
Numbers of patients	2049	636	143			
Number of lesions	2408	778	167			
Age (years)	68.6 ± 10.9	70.7 ± 9.0	67.3 ± 12.0	< 0.0001	0.156	< 0.0001
Body mass index	26.8 ± 4.1	28.0 ± 4.6	28.3 ± 4.6	< 0.0001	0.001	0.797
Female gender (%)	1088 (45.2)	343 (44.1)	72 (43.1)	0.547	0.365	0.575
Arterial hypertension (%)	1883 (78.2)	712 (91.5)	145 (86.8)	< 0.0001	0.013	0.036
Smokers (%)	1183 (49.1)	377 (48.5)	88 (52.7)	0.737	0.261	0.224
Dyslipidaemia (%)	1532 (63.7)	587 (75.8)	109 (65.3)	< 0.0001	0.870	0.002
Chronic kidney disease (%)	418 (17.4)	200 (25.7)	38 (22.8)	< 0.0001	0.036	0.591
LV ejection fraction (%)	54.8 ± 9.6	53.0 ± 11.2	53.9 ± 11.6	< 0.0001	0.304	0.281
Target organ damage^§^ (%)	1359 (56.4)	558 (71.6)	133 (79.2)	< 0.0001	< 0.0001	0.046
Previous PCI (%)	821 (36.4)	268 (36.7)	59 (38.8)	0.866	0.389	0.468
ACS (%)	888 (37.2)	249 (32.3)	46 (28.4)	0.013	0.014	0.246
LAD (%)	1544 (64.1)	485 (62.3)	112 (67.1)	0.359	0.499	0.283
Proximal segments (%)	1397 (60.7)	460 (61.7)	86 (56.6)	0.632	0.274	0.208
Multivessel disease (%)	864 (35.9)	344 (44.2)	76 (45.5)	< 0.0001	0.006	0.591
Diameter Stenosis (%)	58.5 ± 11.0	59.1 ± 10.9	58.2 ± 11.7	0.197	0.527	0.226
FFR	0.84 ± 0.08	0.83 ± 0.07	0.85 ± 0.08	0.034	0.921	0.380
iFR	0.90 ± 0.10	0.88 ± 0.10	0.89 ± 0.12	0.263	0.456	0.951
Pd/Pa	0.93 ± 0.05	0.92 ± 0.04	0.92 ± 0.05	0.275	0.067	0.215
Discordance FFR/NHPRs*	58 (19.4)	22 (20.6)	6 (13.3)	0.202	0.087	0.081
Abnormal FFR (%)	663 (30.8)	208 (31.8)	49 (37.1)	0.658	0.103	0.191
Abnormal NHPR (%)	325 (31.1)	121 (35.2)	41 (44.1)	0.167	0.008	0.098
Abnormal Physiology	755 (31.5)	248 (32.2)	63 (39.1)	0.744	0.036	0.074
Deferred lesions (%)	1666 (69.2)	539 (69.3)	118 (70.6)	0.977	0.563	0.578

*IDDM* insulin dependent diabetes mellitus, *DM* diabetes mellitus, *CKD* chronic kidney disease, *LV* left ventricle, *ACS* acute coronary syndrome, *LAD* left anterior descending, *FFR* fractional flow reserve, *iFR* instantaneous wave free ratio, *NHPR* non-hyperemic pressure ratio

^*^Lesions with both FFR and NHPR available

^§^Target organ damage was defined as eGFR < 30 ml/min/1.73 m^2^ and/or severe target organ vasculopathy (including multivessel coronary disease, carotid artery disease or peripheral vascular disease)

### Primary endpoint

**Fig. 2:**
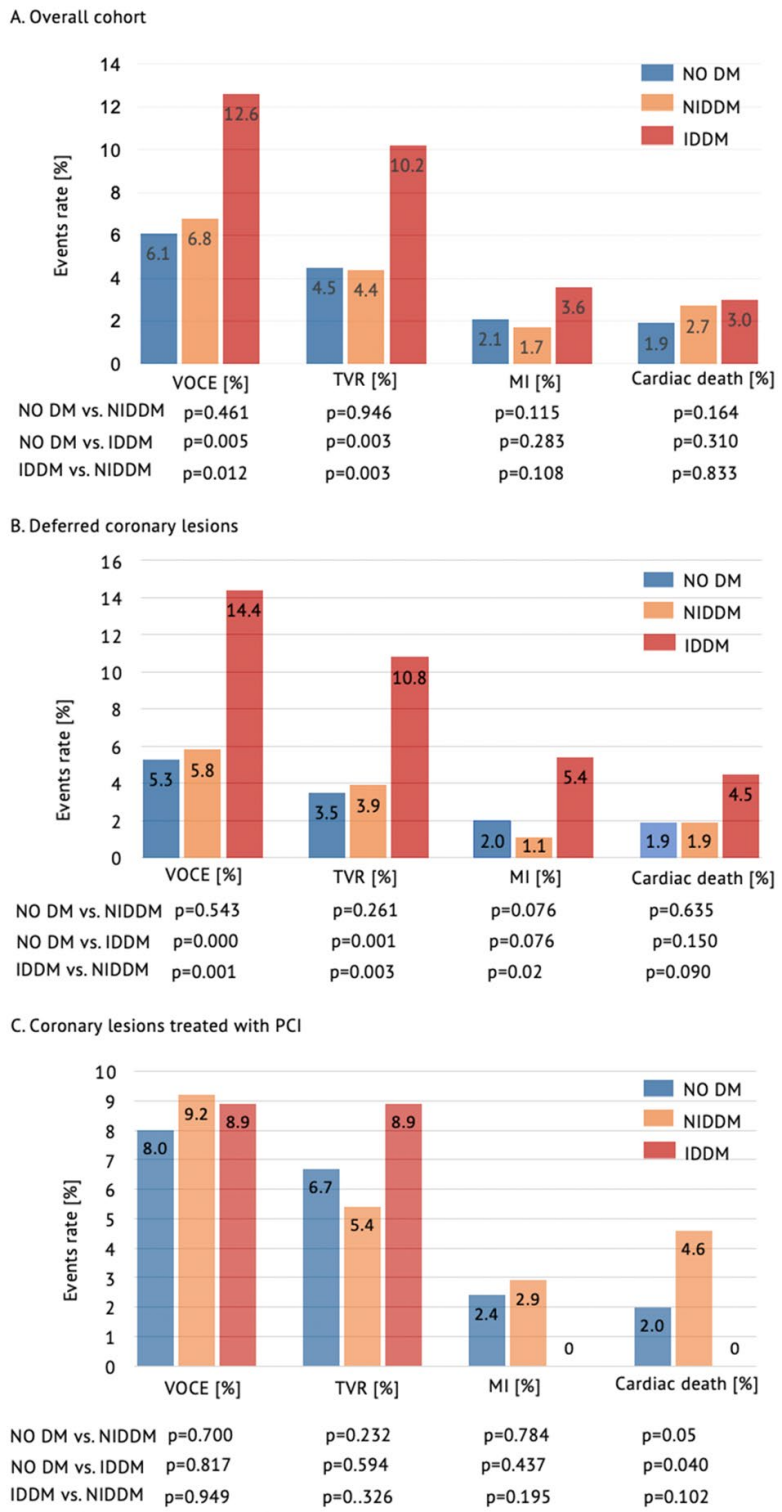
Adverse Events Rate. Primary and secondary endpoints in overall cohort (**A**) and in patients with coronary lesions deferred (**B**) or treated with PCI (**C**)

During the follow-up time, the primary endpoint occurred in 222 (6.6%) coronary lesions, including 159 (4.7%) ischemia-driven TVR, 70 (2.1%) vessel-oriented MI and 72 (2.1%) cardiac death. Patients with IDDM showed a twofold higher rate of VOCE compared with patients without DM ((12.6% vs. 6.1%, *P* = 0.005) and patients with NIDDM (12.6% vs. 6.8%, *P* = 0.012, Fig. 2).

**Fig. 3:**
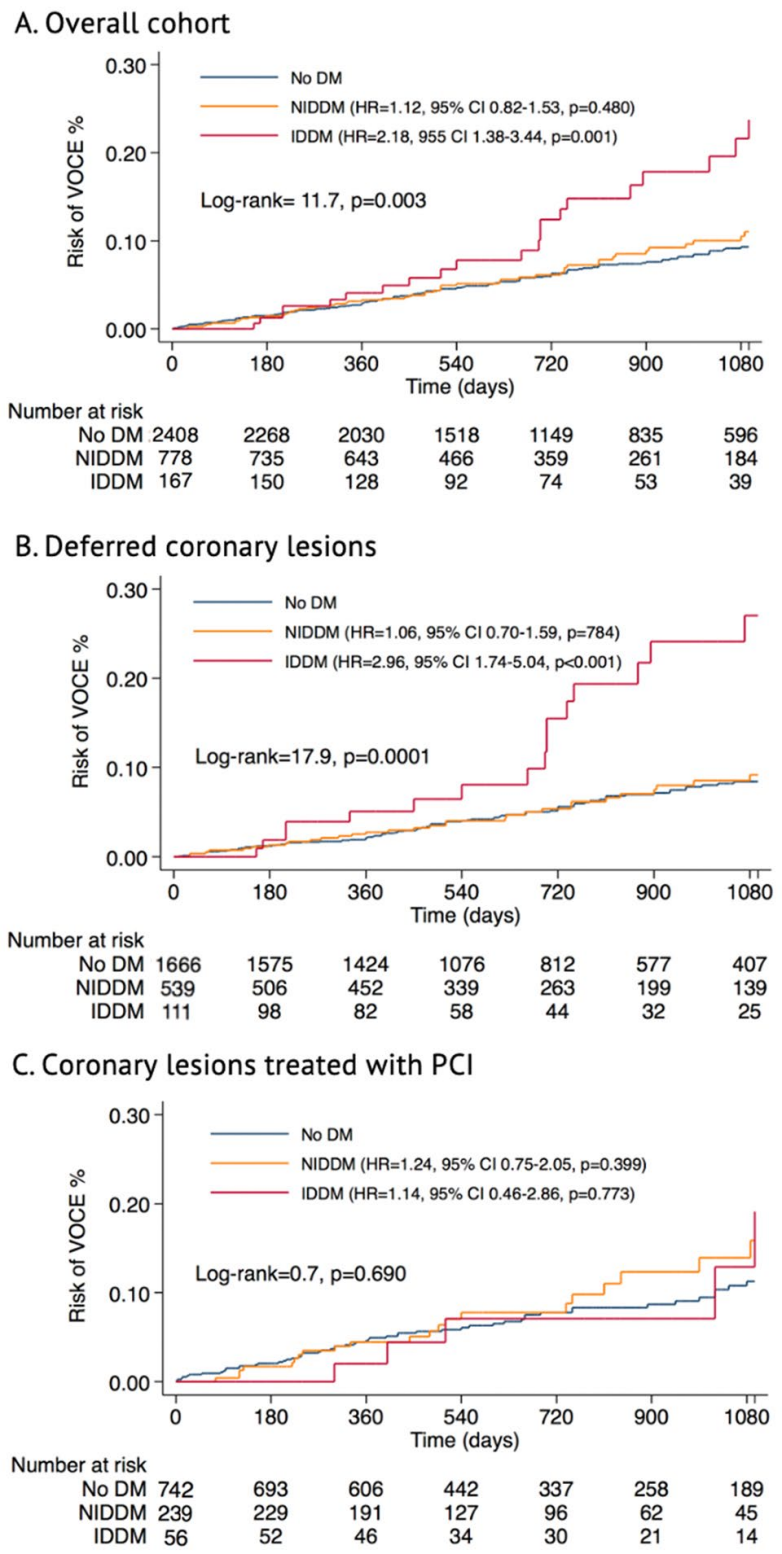
Survival analysis. Risk of VOCE in the overall cohort (**A**) and in patients with coronary lesions deferred (**B**) or treated with PCI (**C**)

Among patients with DM, NIDDM was not associated with the primary endpoint (HR 1.04, 95% CI 0.76–1.42, *P* = 0.782, Table 2, Fig. 3). Conversely, IDDM was independently associated with VOCE (aHR 1.76, 95% CI 1.07–2.91, *P* = 0.027, Table 2, Fig. 3). A sensitivity analysis performed considering only coronary lesions assessed with FFR (*n* = 2968 lesions, 88.5%) confirmed these results (Supplemental Table 2). IDDM (*P* for interaction < 0.001) and DM complicated by target organ damage (*P* for interaction = 0.040) but not NIDDM (*P* for interaction = 0.640) determined a significant effect modification in the FFR-based risk stratification.

**Table 2:** Univariable and multivariable Cox regression analysis of the primary endpoint in the overall cohort

	HR (95% CI)	*P*-value	aHR (95% CI)	*P*-value	aHR (95% CI)^†^	*P*-value
Age	1.01(0.99–1.02)	0.408				
Female gender	0.82 (0.63–1.08)	0.160				
Dyslipidaemia	1.34 (1.00–1.81)	0.054	1.30 (0.94–1.80)	0.117	0.94 (0.50–1.77)	0.860
Arterial hypertension	1.48 (1.01–2.18)	0.046	1.38 (0.90–2.11)	0.135	2.01 (0.81–4.99)	0.131
Smoking	0.90 (0.69–1.17)	0.416				
NIDDM§	1.04 (0.76–1.42)	0.782				
IDDM	2.07 (1.32–3.25)	0.002	1.76 (1.07–2.91)	0.027	3.02 (1.23–7.44)	0.016
Chronic kidney disease	1.25 (0.91–1.70)	0.167	1.31 (0.93–1.86)	0.127	2.21 (1.17–4.19)	0.015
Target organ damage*	1.38 (1.02–1.87)	0.034				
LV ejection fraction	0.99 (0.98–1.01)	0.239				
Previous PCI (%)	1.26(0.96–1.66)	0.096				
ACS	1.40 (1.07–1.83)	0.013	1.31 (0.98–1.76)	0.070	1.03(0.57–1.86)	0.921
Multivessel disease	1.17 (0.90–1.53)	0.247				
LAD	1.43 (1.07–1.92)	0.016	1.38 (0.98–1.94)	0.069		
Proximal segments	1.61 (1.19–2.17)	0.002	1.55 (1.12–2.15)	0.008	1.86 (1.04–3.33)	0.036
Diameter Stenosis	1.01(1.00–1.02)	0.105				
FFR	0.03 (0.01–0.14)	0.000				
iFR	0.81 (0.10–10.99)	0.871				
Abnormal Physiology	1.79 (1.37–2.34)	0.000	1.59 (1.19–2.14)	0.002	1.40 (0.80–2.46)	0.237
Abnormal FFR	1.77 (1.34–2.35)	0.000				
Abnormal NHPR	1.46 (0.99–2.15)	0.056				
FFR/NHPRs discordance	1.30 (0.57–2.98)	0.535				

*IDDM* insulin dependent diabetes mellitus, *DM* diabetes mellitus, *CKD* chronic kidney disease, *LV* left ventricle, *ACS* acute coronary syndrome, *LAD* left anterior descending, *FFR* fractional flow reserve, *iFR* instantaneous wave free ratio, *NHPR* non-hyperemic pressure ratio

^†^Multivariable shared frailty Cox regression model, including patient identification, in patients with multivessel disease

^§^ NIDDM was included in a separate multivariable Cox regression model (aHR = 1.18 [0.87–1.59], *p* = 0.276) and shared frailty Cox regression model (aHR = 1.28 [0.71–2.29), *p* = 0.411)

^*^Target organ damage was included in a separate Cox regression model to avoid multicollinearity (aHR = 1.28 [0.93–1.76], *p* = 0.127)

### Secondary endpoints

Predictors of secondary endpoints in the overall cohort are displayed in Supplemental Tables 3, 4 and 5. NIDDM was not significantly associated with any of the individual components of the primary endpoint. IDDM was independently associated with ischemia-driven TVR (aHR 2.13, 95% CI 1.22–3.72, *P* = 0.008, Supplemental Table 2), but not with vessel-oriented MI and cardiac death (Supplemental Figs. 2–4A).

### Predictors of VOCE in deferred coronary lesions

Deferral rate was not different in coronary lesions of patients without DM (69.2%), patients with NIDDM (69.3%) and patients with IDDM (70.6%) (Table 1). Seventy-nine lesions (3.4%) were deferred despite abnormal coronary physiology findings. Patients in this subgroup presented less frequently with ACS (21.8% vs 33.9%, *P* = 0.028) and they showed higher rates of comorbidities including DM (43.0% vs 27.5%, *P* = 0.005), chronic kidney disease (CKD) (34.2% vs 19.7%, *P* = 0.004) and MVD (54.3% vs 37.5%, *P* = 0.006) (Supplemental Table 6). Operators’ rationale for deferring lesions despite positive FFR/NHPR are reported in Supplemental Table 7 and included mainly distal localization, diffuse disease, severe CKD, and technical complexity.

Overall, VOCE occurred in 136 (5.9%) deferred lesions. After adjustment for clinical confounders, lesion localization in the proximal segment of the coronary artery (aHR 2.20, 95% CI 1.33–3.63, *P* = 0.002), abnormal coronary physiology (FFR ≤ 0.80 or NHPR ≤ 0.89) (aHR 5.95, 95% CI 2.27–15.59, *P* < 0.0001) and IDDM (aHR 2.77, 95% CI 1.11–6.93, *P* = 0.029) were independently associated with the risk of VOCE in the shared frailty Cox regression model (Table 3, Fig. 3). Conversely, NIDDM was not associated with the primary endpoint (HR = 1.06, 95% CI 0–70-1.59, *P* = 0.784; Table 3). Consistently, IDDM determined an effect modification in the FFR-based risk stratification (*P* for interaction < 0.001), contrary to NIDDM (*P* for interaction = 0.426; Central Figure) or DM complicated by target organ damage (*P* for interaction = 0.096). Predictors of secondary endpoints in deferred coronary lesions are displayed in Supplemental Tables 8–10 and Supplemental Figs. 2–4B.

**Table 3:** Univariable and multivariable Cox regression analysis of primary endpoint in deferred coronary lesions

	HR (95% CI)	*P*-value	aHR (95% CI)^§^	*P*-value
Age	1.00 (0.99–1.02)	0.755		
Female gender	0.72 (0.51–1.02)	0.062	0.56 (0.35–0.95)	0.029
Dyslipidaemia	1.27 (0.87–1.85)	0.221		
Arterial hypertension	1.63 (0.98–2.71)	0.059		
Smoking	0.90 (0.64–1.27)	0.545		
NIDDM^†^	1.06 (0.70–1.59)	0.784		
IDDM	2.92 (1.73–4.94)	< 0.0001	2.77 (1.11–6.93)	0.029
Chronic kidney disease	1.32 (0.90–1.95)	0.158	1.28 (0.70–2.33)	0.415
Target organ damage	1.38 (0.94–2.03)	0.096		
LV ejection fraction	0.99 (0.97–1.00)	0.107		
Previous PCI	1.01(0.70–1.44)	0.969		
Diameter Stenosis	1.01 (0.99–1.02)	0.565		
FFR	0.01 (0.00–0.40)	0.013		
iFR	0.09 (0.00–2.55)	0.158		
Abnormal Physiology	2.99 (1.61–5.55)	0.001	5.95 (2.27- 5.59)	< 0.0001
ACS	1.38 (0.98–1.94)	0.067	1.46 (0.89–2.40)	0.137
Multivessel disease	1.43 (1.02–2.00)	0.040	1.26 (0.75–2.14)	0.375
LAD	1.27 (0.90–1.81)	0.174		
Proximal segments	1.74 (1.18–2.57)	0.005	2.20 (1.33–3.63)	0.002

*IDDM* insulin-dependent diabetes mellitus, *CKD* chronic kidney disease, *LV* left ventricle, *ACS* acute coronary syndrome, *LAD* left anterior descending, *FFR* fractional flow reserve, *iFR* instantaneous wave-free ratio, *NHPR* non-hyperaemic pressure ratio

^§^Multivariable shared frailty Cox regression model, including patient identification

^†^NIDDM was included in a separate multivariable shared frailty Cox regression model. Adjusted HR was 0.94(0.61–1.44), *p* = 0.776

### Predictors of VOCE in coronary lesions treated with PCI

PCI was performed more frequently in patients presenting with ACS (40.1% vs. 33%, p < 0.001), lesion localization in the LAD (79.3% vs. 57%, *P* < 0.001) and in the proximal segments of the coronary vessels (61.7% vs. 56.3%, *P* = 0.018) and less frequently in patients with previous PCI (31.5% vs. 35%, *P* = 0.006) compared with the deferred group (Supplementary Table 11). In this subgroup, VOCE occurred in 8.3% of the cases, without significant differences between patients without DM and patients with NIDDM and IDDM, Fig. 2 C, (Log-rank = 0.7, *P* = 0.690, Fig. 3C). At Cox regression analysis, previous PCI (aHR 1.90, 95% CI 1.22–2.96, *P* = 0.004) was the only variable independently associated with the risk of VOCE (Table 4). NIDDM (p for interaction = 0.755), IDDM (*P* for interaction = 0.362) and DM complicated by target organ damage (*P* for interaction = 0.242) did not determine significant effect modification in the FFR-based risk stratification in coronary lesion treated with PCI. Predictors of the secondary endpoints in this subgroup are displayed in Supplemental Tables 12–14.

**Table 4:** Univariable and multivariable Cox regression analysis of primary endpoint in coronary lesions treated with PCI

	HR (95% CI)	*P*-value	aHR (95% CI)^§^	*P*-value
Age	1.01 (0.99–1.03)	0.221		
Female gender	1.11 (0.71–1.71)	0.649		
Dyslipidaemia	1.47 (0.90–2.39)	0.135		
Arterial hypertension	1.46 (0.78–2.76)	0.238		
Smoking	0.83 (0.54–1.26)	0.384		
NIDDM	1.24 (0.75–2.05)	0.399	1.30 (0.78–2.16)	0.314
IDDM	1.14 (0.46–2.86)	0.773	1.26 (0.50–3.16)	0.621
Chronic kidney disease	1.05 (0.61–1.81)	0.872	0.91 (0.51–1.60)	0.739
Target organ damage	1.37 (0.84–2.24)	0.204		
LV ejection fraction	1.00 (0.98–1.03)	0.683		
Previous PCI	1.90 (1.22–2.95)	0.004	1.90 (1.22–2.96)	0.004
Diameter Stenosis	1.00 (0.98–1.02)	0.871		
ACS	1.27 (0.82–1.96)	0.278		
Multivessel disease	0.81 (0.51–1.28)	0.380	0.87 (0.54–1.38)	0.553
LAD	1.44 (0.80–2.61)	0.222		
Proximal segments	1.43 (0.88–2.34)	0.148	1.47 (0.89–2.41)	0.132
Stent length	0.99 (0.96–1.01)	0.284		

*IDDM* insulin-dependent diabetes mellitus, *DM* diabetes mellitus, *CKD* chronic kidney disease, *LV* left ventricle, *ACS* acute coronary syndrome, *LAD* left anterior descending, *FFR* fractional flow reserve, *iFR* instantaneous wave-free ratio, *NHPR* non-hyperaemic pressure ratio, *NIDDM* non-insulin-dependent DM

^§^Multivariable shared frailty Cox regression model, including patient identification. Variables with *p*-value < 0.1 at univariable analysis and variables considered a priori associated with VOCE were included in the multivariable model

Comparing the vessel-oriented outcome of patients who underwent PCI vs those with deferred coronary lesions, deferral was associated with a lower risk of VOCE in patients without DM (HR = 0.70, 95% CI 0.50–0.97, *P* = 0.033) and a trend towards lower events in NIDDM (HR = 0.58, 95% CI 0.33–1.02, *P* = 0.057). No significant difference was observed between lesions treated vs deferred in patients with IDDM (HR = 1.88, 95% CI 0.69–5.15, *P* = 0.217). (Supplementary Fig. 5).

### Coronary physiology assessment in patients with diabetes mellitus

The angiographic CAD severity was similar between coronary lesions of patients without DM, patients with NIDDM and patients with IDDM (Table 1). However, patients with NIDDM showed lower values of FFR compared with patients without DM (0.83 ± 0.07 vs 0.84 ± 0.08, *P* = 0.034). Moreover, the rate of abnormal coronary physiology was higher in patients with IDDM compared with patients without DM (39.1% vs 31.5%, *P* = 0.036).

In patients with diabetes and deferred coronary lesions, lesion localization on the left anterior descending artery (aHR 3.13, 95% CI 1.31–7.51, *P* = 0.010) and IDDM (aHR 2.47 95% CI 1.29–4.73, *P* = 0.006) were associated with increased risk of VOCE after adjustment for clinical confounders (Supplemental Table 15). IDDM was an independent predictor of ischemia-driven TVR (aHR 2.18, 95% CI 1.16–4.11, *P* = 0.016) and vessel-oriented MI (aHR 3.43, 95% CI 1.05–11.23, *P* = 0.042) but not of cardiac death (Supplemental Table 16–18).

## Discussion

We have reported data on long-term clinical outcome of a large, multicentre, all-comers cohort of patients with and without DM who underwent coronary physiology-guided coronary revascularization. The main results of this analysis are the following:

1. NIDDM is not independently associated with VOCE in coronary vessels functionally evaluated with wire-based coronary physiology.

2. NIDDM does not cause significant effect modification of FFR risk stratification and it is not associated with increased risk of adverse events in lesions deferred after physiological assessment.

3.Patients with insulin-dependent DM are at high risk of VOCE, especially ischemia-driven TVR and target-vessel MI.

The association between DM and cardiovascular adverse events is well known [10–12]. However,the risk of VOCE was not significantly different in patients with and without NIDDM in the overall cohort and in the subgroups of patients with coronary lesions deferred or treated with PCI (Fig. 3, Tables 2, 3 and 4). This is consistent with what was previously observed by other investigators [13]. Nonetheless, the association between IDDM and adverse outcomes after PCI was also previously established. A large meta analysis [14] that included 21,759 patients with DM who underwent PCI, demonstrated a significantly higher rate of adverse events in patients with IDDM compared with patients with non-insulin-treated DM. Consistently, the independent prognostic role of IDDM was recently confirmed in patients who underwent PCI with second-generation drug-eluting stents [15]. In our analysis, IDDM was not associated with vessel-oriented adverse outcomes in coronary lesions treated with PCI. However, patients with IDDM demonstrated a significant excess risk of VOCE especially in the subgroup with deferred coronary lesions (Central Figure).

In a relatively small cohort of 205 patients with DM, of which 87 (42.4%) IDDM, Kennedy et al. [16] demonstrated an association between IDDM and adverse events in coronary lesions deferred based on FFR assessment (HR 2.24, 95% CI1.01–4.95, *P* = 0.046). Our findings confirm and further expand these observations on a much larger cohort of patients with longer-term follow-up. In our study, IDDM resulted in an independent predictor of ischemia-driven TVR and vessel-related MI after coronary physiology-guided revascularization deferral (Supplemental Figs. 2, 3B and Table 8 and 9).

The choice of performing or deferring coronary revascularization was left to the operator’s clinical judgment and 3.4% of the deferred lesions showed abnormal values of coronary physiology. These patients showed more comorbidities, multivessel involvement and angiographically more severe lesions (Supplemental Table 6). Abnormal coronary physiology was strongly associated with adverse clinical outcomes in deferred coronary lesions, as previously demonstrated by landmark trials [17] (Table 3), confirming the continuous association between FFR risk stratification and vessel-related adverse outcomes. This association was not modified by NIDDM. Conversely, IDDM and DM complicated by target organ damage significantly interacted with the FFR-based risk stratification, increasing the risk of VOCE for each value of FFR. IDDM tended to show target organ damage more frequently compared with NIDDM. Indeed, patients with IDDM tend to have long disease history, multiple comorbidities [18] and suboptimal glycaemic control compared with non-insulin-treated DM patients. Moreover, exogenous insulin was previously correlated with atherogenesis, increasing pro-inflammatory macrophage response and fibrinogen production [19, 20]. The oscillations of blood glucose levels observed in IDDM have been demonstrated to be associated with the development of thin cap fibroatheroma, which is linked with spontaneous plaque rupture and adverse clinical events [21]. The “Thin-cap fibroatheroma predicts clinical events in diabetic patients with normal fractional flow reserve” (COMBINE-OCT FFR) Trial [22] demonstrated a significantly higher rate of cardiovascular adverse events at 18 months follow up in patients with coronary lesions with FFR > 0.80 and thin cap fibroatheroma compared with patients without thin cap fibroatheroma. However, the proportion of patients with IDDM was similar in patients with and without thin cap fibroatheroma.

### Safety of physiology-guided coronary revascularization in patients with diabetes

Patients with DM often present multivessel and diffuse coronary disease. In these scenarios, coronary physiology may offer important clinical benefits, changing the interventional strategy in a significant proportion of patients [23]. However, the reliability of intracoronary functional assessment in DM has been questioned based on previous observations of lower hyperaemic myocardial blood flow compared with controls [24]. Indeed, impaired coronary microvascular function and/or endothelial dysfunction may reduce the vasodilatory microcirculatory response to a hyperaemic stimulus and produce a falsely negative FFR [3, 25]. Nonetheless, in this study, the mean value of FFR was lower in patients with NIDDM compared with those without DM (Table 1) despite similar angiographic severity, excluding an overall FFR underestimation. While the majority of the coronary lesions were evaluated using only FFR, NHPRs (mainly iFR) were available in nearly 30% of cases. The rate of VOCE was similar among patients treated according to FFR-guided or NHPR-guided strategy (Supplemental Fig. 6), confirming the observation of a post-hoc analysis of the DEFINE-FLAIR trial [12]. DM has been previously associated with an increased prevalence of FFR/NHPR discordance [26, 27]. However, this was not confirmed by our analysis and FFR/NHPR discordance was not associated with the risk of VOCE.

### Limitations

This study has several limitations. First, this is an observational, retrospective, non-randomized study. Nevertheless, the large sample size provided significant statistical power in assessing the risk of VOCE. Moreover, the multicenter design limited potential bias in the composition of the study cohort. Adverse events were not centrally adjudicated but they were reported by the investigators. Furthermore, a systematic three-vessel coronary physiology assessment was not performed and the choice of which lesion to assess with FFR and/or NHPRs was left to the operator’s discretion. Therefore, we cannot exclude, that lesions not evaluated with pressure-wire may have contributed to determine patients’ outcome. For this reason, we decided to perform the analyses on a per-vessel level, focusing on target vessel adverse events. However, in patients who experienced the primary endpoint at follow-up, it was not possible to distinguish if VOCE were related to the suspected target lesions that underwent physiology assessment during the index procedure or rather to different lesions within the same vessel. The lack of intracoronary imaging, which prevented the evaluation of plaque composition and its correlation with outcomes, must be considered an additional limitation of this study [22, 28–30].

Data regarding medical therapy in patients with DM allowed only the distinction between insulin-dependent vs non-insulin-dependent DM. Therefore, it was not possible to determine the association between medical therapy (other than insulin) and the risk of target lesion failure. Moreover, in this series, the number of IDDM was relatively low compared with other reports. Nonetheless, we were able to show a significant association between IDDM and the risk of VOCE. Chronic glycaemic control and anaemia are important determinants of clinical outcomes in patients with DM presenting with acute and chronic coronary syndromes [31]. However, these data were not available for all the patients and were not included in the analysis. Additionally, other clinical features including retinopathy, proteinuria and left ventricular hypertrophy, were not available and thus could not be included in the definition of target organ damage. If these characteristics are associated with the risk of VOCE in patients undergoing functional coronary assessment must be assessed in future dedicated studies.

## Conclusion

Patients with non-insulin-dependent DM and coronary lesions assessed with coronary physiology demonstrated a low risk of VOCE at long-term follow-up, similar to the risk of patients without DM. Conversely, patients with IDDM represent a subgroup at high risk of vessel-related adverse events and require close monitoring at follow-up, even in the presence of non-ischemic findings at coronary functional assessment.

## Data Availability

Data are available on reasonable request.

## Abbreviations

CAD: Coronary artery disease
CKD: Chronic kidney disease
PCI: Percutaneous coronary intervention
MI: Myocardial infarction
DM: Diabetes mellitus
FFR: Fractional flow reserve
aHR: Adjusted hazard ratio
IDDM: Insulin-dependent diabetes mellitus
iFR: Instantaneous wave free ratio
NIDDM: Non-insulin-dependent diabetes mellitus
NHPR: Non-hyperemic pressure ratio
VOCE: Vessel-oriented cardiac adverse events
TVR: Target vessel revascularization
